# Methods to infer transmission risk factors in complex outbreak data

**DOI:** 10.1098/rsif.2011.0379

**Published:** 2011-08-10

**Authors:** Simon Cauchemez, Neil M. Ferguson

**Affiliations:** MRC Centre for Outbreak Analysis and Modelling, Department of Infectious Disease Epidemiology, Imperial College London, London, UK

**Keywords:** infection disease, epidemiology, statistics, expectation maximization, Markov chain Monte Carlo, mathematical modelling

## Abstract

Data collected during outbreaks are essential to better understand infectious disease transmission and design effective control strategies. But analysis of such data is challenging owing to the dependency between observations that is typically observed in an outbreak and to missing data. In this paper, we discuss strategies to tackle some of the ongoing challenges in the analysis of outbreak data. We present a relatively generic statistical model for the estimation of transmission risk factors, and discuss algorithms to estimate its parameters for different levels of missing data. We look at the problem of computational times for relatively large datasets and show how they can be reduced by appropriate use of discretization, sufficient statistics and some simple assumptions on the natural history of the disease. We also discuss approaches to integrate parametric model fitting and tree reconstruction methods in coherent statistical analyses. The methods are tested on both real and simulated datasets of large outbreaks in structured populations.

## 1. Introduction

Data collected during field outbreak investigations are essential to better understand the clinical and epidemiological features of an infectious disease. They can also provide useful insights for outbreak management and control. For example, evaluating the risk factors governing transmission is important to design efficient control measures, and identify those individuals that are most at risk of infection or are the main contributors of infection and should therefore be targeted first.

However, characterizing transmission from outbreak data can be challenging. First, the transmission process is usually imperfectly observed. For example, we may observe the date of symptoms onset of a case, but we rarely know where, when and by whom a case was infected. Inference, therefore, requires integrating over ‘missing data’, which may quickly become cumbersome. Over the last 15 years, data augmentation methods have been used to tackle this problem: data are augmented with missing data (e.g. dates of infection) that are needed to write down the likelihood; in a Bayesian setting, the joint posterior distribution of parameters and augmented data is explored usually via Markov chain Monte Carlo (MCMC) sampling [1]. This methodology is now well established in the field and has been successfully applied to analyse a range of complex datasets. However, for relatively large outbreaks with detailed data, this approach may require very long computational times. Interested readers can, for example, read references [2–11].

The second challenge is that the type of dependency between observations that is typically observed in an outbreak (i.e. the risk of infection of an individual depends on the infection status of other individuals) is specific to communicable diseases and needs to be accounted for with dedicated methods. This usually requires that the statistical model used to analyse the data is explicitly based on a mechanistic model of disease spread [12]. Transmission parameters of interest, for example, the reproduction number (the number of individuals infected by a case), are usually mathematically defined in those models. Fitting such parametric mechanistic models to outbreak data can give useful insights on transmission [7,13–15], but is subject to the same limitations as parametric fitting in other fields. For example, although there are some exceptions [10], the approach usually requires to predefine time intervals on which transmission rates are constant. This can sometimes be difficult to achieve in a non-ad hoc way.

An alternative approach that has become increasingly popular is to reconstruct the transmission tree and derive important summary statistics from it, for example, the temporal trends in the reproduction number [16–18]. This may give greater flexibility (for example, there is no need to specify time intervals with constant transmission rates), potentially at the cost of larger variance of the estimates [15,18]. However, since these methods generally only consider disease cases rather than the uninfected, but potentially susceptible bulk of the population, they can say little about the risk factors for infection or provide estimates of transmissibility in different contexts (e.g. households, schools or as a function of distance between a susceptible and an infected individual).

Overall, the two methodologies (fitting of a parametric mechanistic model and tree-reconstruction methods) are largely complementary. Fitting a mechanistic model seems to be the only way to account for the depletion of susceptibles, the information on uninfected individuals leading to a quantification of relative risks; and it may ensure a better control of the variance of the estimates. Tree-reconstruction methods can provide further insights on what effectively happened during the outbreak with summary statistics on who was infected by whom, when and where and temporal change in the reproduction number. They can also provide a framework to detect abnormal features in the data that are not initially accounted for in a mechanistic model. It is therefore important that the two approaches can be integrated in a coherent way.

In this paper, we discuss strategies to tackle some of the ongoing challenges in the analysis of outbreak data. We present a relatively generic statistical model for the estimation of transmission risk factors, and discuss algorithms to estimate its parameters for different levels of missing data. We look at the problem of computational times for relatively large datasets and show how they can be reduced by appropriate use of discretization, sufficient statistics and some simple assumptions on the natural history of the disease. We also discuss approaches to integrate parametric model fitting and tree reconstruction methods in coherent statistical analyses. The methods are tested on both real and simulated datasets of large outbreaks in structured populations.

## 2. Transmission model, dependency and computational times

Assume that we observe the spread of a disease in a population of size *N* from day 0 to day *T*. For each individual *i* = 1, … , *N*, let 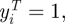 if individual *i* is infected between 0 and *T*; 0 otherwise. Each individual *i* is characterized by a vector of *Q* covariates **z**_***i***_**(*t*)** = {*z*_*i*_^1^(*t*), … , *z*_*i*_^*Q*^ (*t*)} such as age, gender, location, household ID, etc … that may vary with day *t*. We want to quantify the transmission risk factors.

We first consider the situation where day *t*_*i*_ of infection of each case *i* is observed (by convention, *t*_*i*_ = *T* + 1, if individual *i* escaped infection up to day *T*). This assumption is relaxed in §5.

### 2.1. Transmission models

For a directly transmitted disease, the first step to estimate transmission risk factors is usually to propose a model for transmission hazard 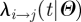 from case *i* to subject *j* on day *t*, i.e. define 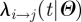 as a function of the individual covariates *z*_*i*_(*t*) and *z*_*j*_(*t*) and a set of parameters *Θ*. For example, the transmission hazard 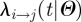 may depend on:

— the time lag *t–t*_*i*_ between day *t* and the day of infection *t*_*i*_ of case *i* since infectiousness of a case may vary during the course of infection. The functional form between 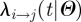 and *t–t*_*i*_ will depend on the assumed natural history of the disease;
— the individual characteristics of subjects *i* and *j*. For example, some subjects may be more infectious while others are more susceptible; and
— the type of interactions that exist between subjects *i* and *j*. The contact rate might, for example, depend on whether the subjects live in the same household or go to the same school, etc. It could also depend on the spatial distance between them.

Examples of specifications for the transmission hazard 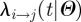 are given in §§6 and 7. The force of infection exerted on individual *j* on day *t* is then the sum:
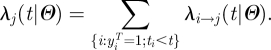


### 2.2. Likelihood and computational time

The contribution to the likelihood of case *j* is:2.1
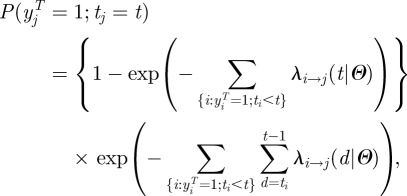
where the first term is the probability of infection on day *t,* and the second term is the probability to escape infection up to day *t* (the link between the continuous time and discrete time transmission model is discussed in appendix A). The contribution of non-case *j* is:2.2
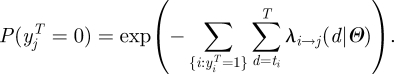


The log-likelihood is therefore:2.3
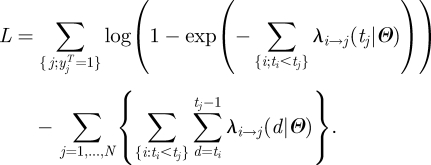


The dependency between observations (i.e. the risk of infection of an individual depends on the infection status of other individuals) that is apparent in equation (2.3) means that computational times required to calculate the likelihood explode with the size of the outbreak. For example, for the 2001 UK foot and mouth disease (FMD) outbreak (about 2000 infected premises (IPs) among 130 000 farms), the number of pairs of farms to be considered per calculation of the likelihood is over 10^8^ [19]. Even with the recent increase in computational power, brute force exploration of the system, though feasible, is very time consuming. Fast and efficient algorithms are necessary to provide real-time support to decision making.

## 3. Discretization and sufficient statistics

Here, we explore the extent to which the discretization of the transmission risk factors can reduce the computational burden associated with the evaluation of equation (2.3). Therefore, we now restrict our analysis to the situation where each transmission risk factor takes a finite set of values. We will then explore how the approach can be used to investigate continuous risk factors.

### 3.1. Discretization

Assume that the transmission hazard between two individuals depends on *K* risk factors ***x*** = {*x*^1^, … ,*x*^*K*^} and that the *k*-th risk factor *x*^*k*^ (*k* = 1, … , *K*) takes a finite number (=*C*_*k*_) of values 
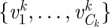
. The set ***Ψ*** of possible values for risk factor vector ***x*** = {*x*^1^, … , *x*^*K*^} has size 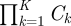. For example, the list of risk factors might include the (discretized) distance between the individuals (either spatial or social, e.g. members of the same household), the time lag since infection or individual characteristics, such as age.

We model the transmission hazard between a case and a susceptible individual in the population by:3.1
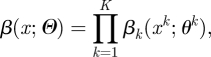
where the specific effect of *x*^*k*^ on the transmission hazard is measured by function 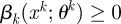, and 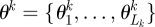 is a parameter vector of size *L*_*k*_. This expression makes the simplifying assumption that the effect of all risk factors on the hazard can be expressed as the product of the impacts of each factor. Parameters of the model are 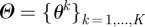.

Remembering that ***z***_***i***_ denotes data available for individual *i*, we assume here that risk factors for transmission from case *i* to individual *j* on day *t* are a function of triplet {***z***_***i***_, ***z***_***j***_, *t*}: 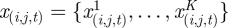, where for *k* = 1, … , *K*, 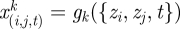.

The transmission hazard 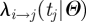 from case *i* to individual *j* on day *t* is therefore:
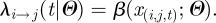


The total hazard of infection for individual *j* on day *t* is then:
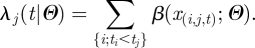


### 3.2. Reduction of the computational burden: sufficient statistics

If transmission risk factors are discretized, equation (2.3) can be re-written:3.2
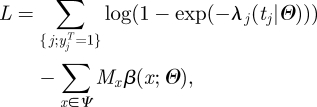
where
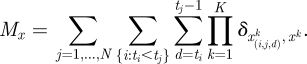


Here *δ*_*a,b*_ is the Kronecker delta or identity function (=1 if *a* = *b* and 0 otherwise). *M*_*x*_ counts the number of day-transmission events of type *x*, which might have occurred before time *t* but did not.

Equation (3.2) has important implications in terms of computational speed since it shows that the computational burden can be substantially reduced without loss of information. The first term of equation (3.2) only involves disease cases; the second term characterizes the probability of escaping infection up to time *t*. So, data needs for inference reduce to:

— ‘case data’;
— table 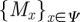 of sufficient statistics that characterize the interaction of cases with any individual of the population. It can be pre-computed and stored once, given they do not functionally depend on the parameters.

## 4. Exploration of the parameter space and tree reconstruction

Tree reconstruction [7,16–18] is a useful complement to the likelihood-based estimates of the parameters derived from equation (3.2). Here, we present and discuss different strategies to perform tree reconstruction and parameter estimation in a coherent statistical framework.

### 4.1. Tree reconstruction

Given parameter *Θ* and given that case *j* was infected on day *t*_*j*_, the probability that case *j* was infected by case *i* (*t*_*i*_ < *t*_*j*_) is simply (see appendix A):4.1
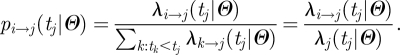


### 4.2. Sequential approach

A natural way to integrate tree reconstruction and parameter estimation in a coherent setting is to proceed sequentially. For example, in a Bayesian setting, a sample 
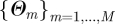
 can be drawn from the posterior distribution of *Θ* via MCMC sampling relying on equation (3.2). Then, for each parameter value in the sample *m* = 1, … ,*M*, a source of infection *r*_*j*_ can be drawn from its distribution 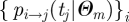 for each case *j*. This gives a sample of *M* transmission trees drawn from their predictive distribution. For example, we used this strategy when analysing detailed data from an influenza outbreak in a school [7].

### 4.3. Simultaneous inference

Here, we explore an alternative strategy where the two tasks are performed simultaneously. The method formalizes those introduced in work undertaken in 2001 on the UK FMD epidemic of that year [13]. The idea is that the source of infection *r*_*j*_ for each case *j* is considered as ‘augmented’ data. The augmented log-likelihood is:4.2
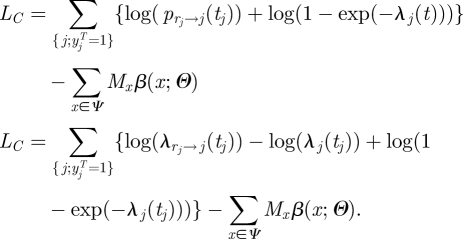


Denoting 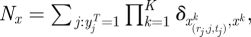 the number of augmented transmission events of type *x* (*x* in *Ψ*), equation (4.2) becomes:4.3
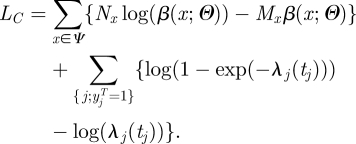


In many situations, where the force of infection exerted on individuals is relatively small, the likelihood simplifies to:4.4
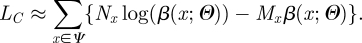


The analysis of the augmented likelihood can be performed in a frequentist or in a Bayesian setting. In the frequentist setting, it is straightforward to implement an expectation conditional maximization (ECM) algorithm [20,21] to both derive maximum-likelihood estimates of the parameters of the model and reconstruct the transmission tree. The pseudo-code for this algorithm is given in box 1. In the common situation, where one is interested in relative risks (i.e. comparison with a reference group) and where the force of infection exerted on individuals is relatively small, no maximization routine is needed since the maximum value is simply a ratio of two sums (appendix A) [13]. Confidence intervals can be obtained from the Fisher information matrix derived at the maximum value for the incomplete log-likelihood (equation 3.2). The approach, therefore, requires that the second derivative of the log-likelihood with respect to *Θ* exists.

Alternatively, inference can be performed in a Bayesian setting via MCMC [1], with a pseudo-code presented in box 2. The key difference is in the way missing data are handled: in the Bayesian approach, one realization of the missing data is drawn from its expected distribution; by contrast in the EM algorithm, the whole expected distribution of the missing data is used in the expression of the augmented likelihood.

Box 1. **The EM algorithm when the days of infection are observed.**

Assume that at the beginning of iteration *n*, parameter vector is *Θ*_*n*__−1_:

— expectation step:
  - for each case *j*, compute probabilities 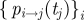 given *Θ*_*n*__−1_ (equation 4.1; see appendix A), and
  - for *x* in *Ψ*, compute the expected number of transmission events of type 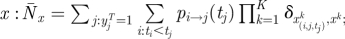 and
— conditional maximization step:
  - for *k* = 1, … , *K*, and
  - for *j* = 1, … , *L*_*k*_: maximize *L*_*C*_ with respect to 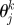 other parameters being fixed (see appendix A):

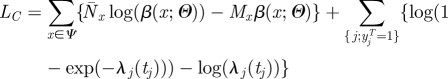

Box 2. **Bayesian algorithm when the days of infection are observed.**

At iteration *n*:

— update missing data:
  - for each case *j*, compute probabilities 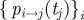 given *Θ*_*n*__−1_ (equation 4.1; see appendix A),
  - for each case *j*, draw the source *r*_*j*_ of case *j* from distribution 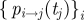, and
  - for *x* in *Ψ*, compute the number of transmission events of type *x*: 
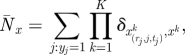
— update parameters:
  - for *k* = 1, … , *K*, and
  - for *j* = 1, … , *L*_*k*_: MCMC update 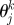 relying on the augmented likelihood *L*_*C*_ (see appendix A):
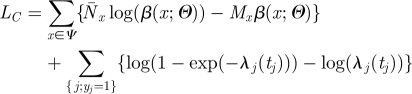

## 5. Situation when the days of infection are unobserved

Often, the day of symptom onset *s*_*j*_ of case *j* is observed but not the day of infection *t*_*j*_. As a consequence, likelihood as shown in equation (3.2) is no longer available. If the incubation period (time lag between the day of infection and the day of symptoms onset) has a known density *f*(*s*_*j*_|*t*_*j*_), a common strategy to tackle the problem is to augment the data with the day of infection of each case. A particular computational burden is then that updating the day of infection of a single case may require re-calculation of the whole likelihood as the update may affect the risk of infection of all other individuals. In order to avoid this computational cost, we introduce the additional assumption:

(H1) Given the day of symptoms onset *s*_*j*_, infectiousness over time is independent of the day of infection *t*_*j*_.

This is, for example, the case if infectiousness starts with symptom onset. This assumption seems acceptable for a relatively wide range of diseases since infectivity is often triggered or influenced by symptoms. Under H1, there is no need to re-compute the whole likelihood each time a day of infection is updated.

It would be possible to extend the EM approach to the situation when the days of infection are unobserved. Inference would work as in the previous section except that one would have to take the expectation on both the contact tracing information and the day of infection. However, for this second application, it is no longer possible to easily derive the variance of the estimates. This is because, although maximum-likelihood point estimates could be derived from the likelihood for the ‘complete’ dataset, estimation of the variance of the estimates has to rely on the likelihood of the observed dataset (equation 3.2). This expression cannot be computed here since days of infection are not observed. The Bayesian approach does not suffer from this limitation and makes it possible to easily obtain Bayesian credible intervals (box 3).

Box 3. **Bayesian algorithm when only days of symptom onset are observed.**

At iteration *n*:

— Gibbs sampling for missing data:
  - for each case *j*, compute probabilities 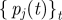 for the day of infection of the case given *Θ*_*n*__−1_ (see appendix A),
  - for each case *j*, draw the day *t*_*j*_ of infection of the case from distribution 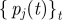,
  - for each case *j*, compute probabilities 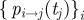 given *Θ*_*n*__−1_ (equation 4.1; see appendix A),
  - for each case *j*, draw the source *r*_*j*_ of case *j* from distribution 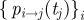, and
  - for *x* in *Ψ*, compute the number of transmission events of type *x*: 
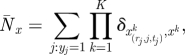
— update parameters:
  - for *k* = 1, … , *K*, and
  - for *j* = 1, … , *L*_*k*_: MCMC update 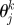 relying on the augmented likelihood. The augmented likelihood is slightly different from the case when days of infection are known (equation 3.2). In particular, since days of infection of cases change during the inference procedure, it is no longer possible to pre-compile and store the contribution of cases to matrix {*M*_*x*_} (i.e. number of day-transmission events of type *x* that could have occurred but did not); but one can still pre-compile and store the contribution {*MM*_*x*_} of non-cases, which is usually the key computational burden. The augmented likelihood is:
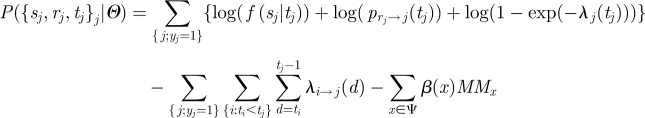

## 6. Application to the foot and mouth disease outbreak with observed days of infection

### 6.1. Data

We reanalyse the 2001 UK FMD dataset presented by Chi-Ster & Ferguson [19]. The dataset contains information on 131 243 farms of which 2013 were IPs, between 7 February 2001 and 5 October 2001. For each farm, the dataset contains the location of the farm and the number of cattle and sheep in each farm. In addition, the data give the estimated time of infection of the farm (if infected) and the removal time (when animals of the farm were slaughtered). Details on the data can be found in Chi-Ster & Ferguson [19].

### 6.2. Specification of the statistical model

We explore how the discretization of risk factors and the algorithms introduced in §4 can reduce the computational burden of inference. For simplicity, farms are partitioned into three groups on the basis of the number of cattle *n*_c_ and the number of sheep 3*n*_s_: cattle (cattle farm: *n*_c_ ≥ *n*_s_), sheep (sheep farm: *n*_s_ > *n*_c_) and small (small farm: *n*_c_ + *n*_s_ < 100) [13]. We assume that the latent period of FMD is 3 days and that infectious farms remain so until the time of slaughter. The transmission hazard *β* between case farm *i* and susceptible farm *j* that is introduced in equation (3.1) is modelled as a function of the following characteristics:

— the type (cattle, sheep or small) of case farm *i*. We estimate the relative infectivity of sheep farms 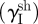 and small farms 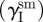 relative to cattle farms. So the multiplicative term on the transmission hazard is 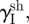 if case farm is a sheep farm, 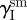 if it is a small farm and 1 if it is a cattle farm;
— the type (cattle, sheep or small) of susceptible farm *j*. We estimate the relative susceptibility of sheep farms 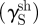 and small farms 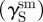 relative to cattle farms; and
— the distance *d*_*ij*_ between farm *i* and farm *j*. Two models are considered: *M1: discrete model*. We assume that the transmission kernel is a step function with *K* + 1 change points {*d*_*k*_}_*k*__=0, … ,__*K*_ and where the multiplicative term on the transmission hazard is 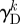 if *d*_*k*__−1_< *d*_*ij*_ ≤ *d*_*k*_. In practice, we take 18 change points {0, 0.5, 1, 1.5, 2, 3, 4, 5, 6, 10, 15, 20, 40, 60, 100, 200, 400, 10^4^} (km). *M2: ‘parametric’ step function*. A continuous transmission kernel often used in the field is6.1
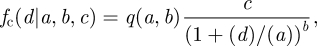
where 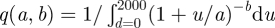 is a normalizing constant. Here, we introduce a discretized version of this kernel. Consider *K* + 1 change points {*d*_*k*_}_*k*__=0, … ,__*K*_. We define 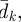 the mean distance between farms *i* and *j* satisfying *d*_*k*__−1_< *d*_*ij*_ ≤ *d*_*k*_:
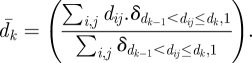
The transmission kernel is the step function:
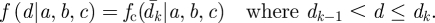
In practice, interval 0–2000 km is partitioned in 250 intervals of length 0.25, 0.5, 1, 2, 5, 10, 20 and 40 km between 0–5, 5–15, 15–65, 65–165, 165–415, 415–815, 815–1015 and 1015–1415 km, respectively.

For the ECM algorithm, we consider that convergence is achieved at iteration *n* if the relative change in parameter values between iteration *n* and iteration *n* + 1 is smaller than 10^−7^ for all parameters. In the Bayesian implementation of the model, we specify the following priors for our parameters. Parameters 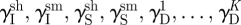 all have a gamma prior *Γ*(*α*, *α*) with *α* = 10^−2^. We also do a sensitivity analysis for *α* = 10^−1^, 1. For model *M2*, we specify a uniform prior *U*[0,100] for kernel parameters *a* and *b* and a gamma prior *Γ*(*α*, *α*) for parameter *c*. The MCMC is run for 8000 iterations with a burn in of 500 iterations.

Transmission parameters are estimated for time interval 23rd February (when the national ban on animal movements was introduced) to 5th October 2001, conditional on the state of the epidemic on 23rd February. Computation times are given for single threaded code running on an Intel Xeon x5570 system.

### 6.3. Results

**Figure 1.**
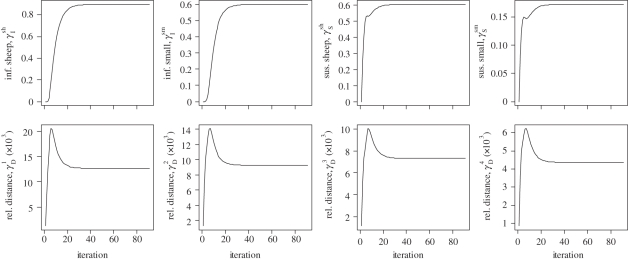
Convergence of the ECM algorithm for model M1.

We first use the ECM algorithm (box 1) to estimate model *M1* (table 1). Convergence is achieved in only 91 iterations (figure 1). Total computational time is 1 min 14 s with most of the time (58 s) spent reading the data and computing the table {*M*_*x*_}_*x*__∈*Ψ*_ of sufficient statistics (equation 3.2). In particular, there is no need to use maximization routines since there is an analytical solution to the conditional maximization step (see appendix A). Computational times are also very short (4 min 28 s) for the Bayesian algorithm (box 2).

**Table 1.** Estimates of relative infectivity and susceptibility of sheep and small farms relative to cattle farms for model *M1* with the EM and the MCMC algorithm.

	EM	MCMC		
		*α* = 0.01	*α* = 0.1	*α* = 1
infectivity				
sheep farm	0.90[0.75,1.04]	0.90[0.76,1.06]	0.89[0.76,1.05]	0.86[0.73,1.00]
small farm	0.60[0.46,0.74]	0.60[0.48,0.74]	0.59[0.47,0.74]	0.57[0.45,0.71]
susceptibility				
sheep farm	0.60[0.54,0.66]	0.60[0.54,0.67]	0.60[0.54,0.67]	0.60[0.54,0.66]
small farm	0.17[0.15,0.19]	0.17[0.15,0.19]	0.17[0.15,0.19]	0.17[0.15,0.19]

Estimates of transmission kernel *M2* are obtained in 6 min 41 s (table 2). Those computational times contrast with those needed through brute calculation of the likelihood (equation 2.3). Replacing model *M2* by the exact continuous parametric kernel (equation 6.1) and using equation (2.3) does not affect estimates (table 2); but computational times move to a month for the same number of iterations of the MCMC, on the same machine and with no serious attempt to optimize the code. While algorithmic optimization and parallel programming allows this to be reduced to a few days [19], the algorithms presented here still give comparable estimates two orders of magnitude more rapidly than brute force approaches.

**Table 2.** Estimates of relative infectivity and susceptibility of sheep and small farms relative to cattle farms, parameters of the transmission kernel for discretized kernel, *f* and the continuous kernel, *f*_c._

	discretized kernel, *f*			continuous kernel, *f*_c_ and brute force inference
	*α* = 0.01	*α* = 0.1	*α* = 1	*α* = 0.01
infectivity				
sheep farm	0.89[0.75,1.05]	0.89[0.76,1.05]	0.89[0.75,1.05]	0.89[0.77,1.05]
small farm	0.59[0.47,0.74]	0.59[0.47,0.74]	0.59[0.47,0.74]	0.59[0.47,0.75]
susceptibility				
sheep farm	0.60[0.54,0.67]	0.60[0.54,0.67]	0.60[0.54,0.67]	0.60[0.54,0.66]
small farm	0.17[0.15,0.19]	0.17[0.15,0.19]	0.17[0.15,0.19]	0.17[0.15,0.19]
kernel par.				
a	1.79[1.4,2.17]	1.78[1.43,2.21]	1.78[1.47,2.17]	1.75[1.43,2.11]
b	2.64[2.5,2.76]	2.64[2.51,2.77]	2.63[2.52,2.76]	2.62[2.51,2.75]
c	0.08[0.07,0.08]	0.08[0.07,0.08]	0.08[0.07,0.08]	0.08[0.07,0.08]

## 7. Simulation study with unobserved days of infection

### 7.1. Simulations

We simulate an epidemic in a city that is structured in households and hospitals and where community transmission can happen. Table 3 summarizes the structure of the city. We consider a city of size 400 000 with an average household size of 2.2 persons and with household demographics consistent with the 1999 French census [22]. The city has three hospitals with 2240 staff members and 800 beds each, a bed occupancy of 70 per cent outside the epidemic period for a duration of hospitalization of 10 days.

**Table 3.** Description of the virtual city in which the epidemic is simulated.

number of inhabitants	400 000
average household size	2.2
number of hospitals	3
number of beds in hospital	800 beds
number of staff per hospital	2240
hospital occupancy outside the epidemic period	70%
duration of hospital visit (days)	10

We simulate the spread of a disease in this population and would like to assess how the techniques described above can be used to evaluate and monitor transmission in the different settings (community, hospital and household), infectivity and susceptibility of different types of individuals (here: children versus adults) along with the efficacy of the interventions that are put in place in the different settings. We are interested in a scenario like the severe acute respiratory syndrome (SARS) rather than, for example, an influenza scenario; that is a disease for which it is possible to detect and identify a substantial proportion of cases.

We assume that the incubation period of the disease has a geometric distribution (with probability 0.3, truncated after 10 days); that individuals start to be infectious on the day of symptoms onset with an infectivity profile following that time which has an exponential shape with mean 3 days (truncated after 20 days). We assume that 20 per cent of cases are hospitalized with equal probability of hospitalization occurring 1 or 2 days after symptoms onset. We assume that children are 1.5-fold more susceptible and more infectious than adults. Following Cauchemez *et al*. [8], we assume that the person-to-person household transmission rate is inversely proportional to the size of the household. The epidemic starts with five cases infected on day 0. Control measures targeting community, household and hospital transmission each with an efficacy of reducing transmission of 50 per cent are implemented on day 60 of the outbreak.

We consider different scenarios for the proportion of cases that are detected in the population. Initially, we assume that all cases are detected. In alternative scenarios, we investigate the situations where 50 or 25 per cent of cases are randomly detected in the community/hospital, but where follow-up of households with detected cases is good (90%) and, last, the situation where detection of cases among household members is of the same quality as detection of cases in the community and in hospitals.

### 7.2. Specification of the statistical model

The transmission hazard *β* between case *i* and individual *j* that is introduced in equation (3.1) depends on the following characteristics:

— setting, i.e. whether individuals *i* and *j* are (i) members of the same household, (ii) have visited the same hospital or (iii) other (i.e. community transmission)—the multiplicative term on the transmission hazard is *β*_Hous_/*n* (*n*: size of the household), *β*_Hosp_/*N*_Hosp_ (*N*_Hosp_: number of staff members plus average hospital occupancy outside an epidemic) and *β*_Com_/*N*_Com_ (*N*_Com_: size of the city). We specify a gamma prior *Γ*(10^−5^, 10^−3^) for *β*_Hous_, *β*_Hosp_ and *β*_Com_;
— infectivity profile from symptom onset of case *i*—modelled with a normalized discretized exponential distribution with a mean to be estimated. We specify a uniform prior *U* [1,10] for the mean value;
— whether or not case *i* is a child—we estimate the infectivity of children relative to adults. We specify a lognormal prior distribution with log-mean 0 and log-variance 1 for the relative infectivity of children *γ*_I_. This ensures that *γ*_I_ has the same prior as 1/*γ*_I_;
— whether or not individual *j* is a child—we estimate the susceptibility of children relative to adults, *γ*_S_, assuming a lognormal prior distribution with log-mean 0 and log-variance 1; and
— efficacy of interventions implemented in the different settings (i.e. household, hospital and community). After implementation of the intervention (day 60), the transmission rate in the household, hospital and community is multiplied by parameter *γ*_Hous_, *γ*_Hosp_ and *γ*_Com_, respectively; where *γ*_Hous_, *γ*_Hosp_ and *γ*_Com_ have the same prior as the relative infectivity and susceptibility of children.

### 7.3. Results

**Figure 2.**
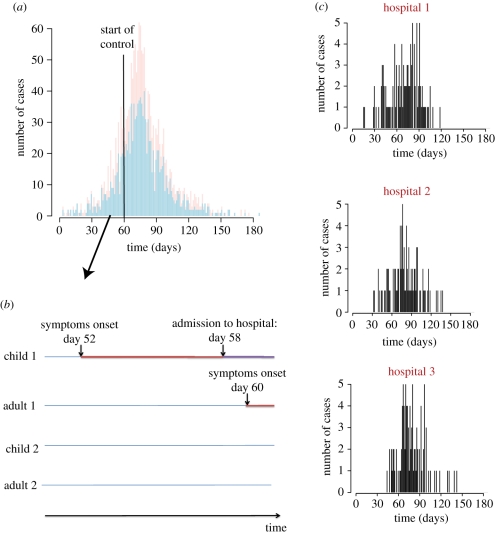
Simulated epidemic and information collected during the epidemic. (*a*) Epidemic curve. (*b*) Follow-up of households. (*c*) Follow-up of outbreaks in hospitals. Pink, child; light blue, adult.

Figure 2 summarizes the data that would need to be collected during the outbreak with the age and dates of symptom onset of cases (figure 2*a*), a follow-up of households with cases and tracking of hospitalizations and more generally of hospital occupancy (figure 2*b*) and a follow-up of epidemics in hospitals (figure 2*c*). Inference also requires having information on the age distribution of the population. In the simulated outbreak, there were a total of 1842 cases with 631 (34%) child cases.

**Figure 3.**
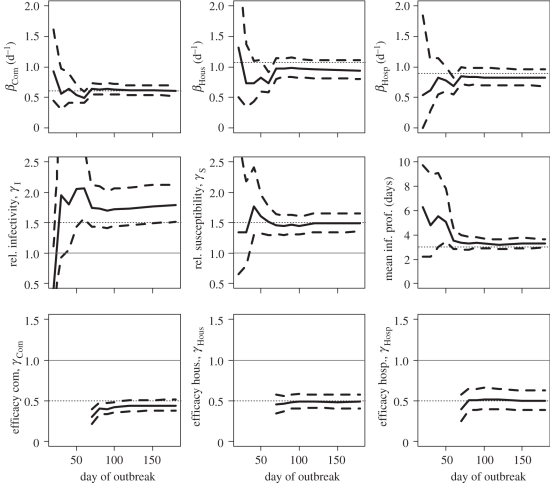
Estimates of transmission rates and relative transmission risk factors as a function of the number of days since the outbreak started when all cases are detected. Solid line, posterior mean; dashed line, 95% Credible Interval; dotted line, simulation value. For parameters used to compare groups (e.g. relative susceptibility, efficacy of interventions, etc.), we have also added a thin horizontal line *y* = 1. Top row gives estimates of the transmission rates in the different settings. Middle row gives estimates of the relative infectivity and relative susceptibility of children and the mean duration characterizing the infectivity profile. Bottom row gives the estimates of the efficacy of intervention to reduce transmission rates in the different settings.

In the scenario, where all cases are identified, figure 3 shows how estimates change in real-time. On day 20, only 31 cases have been detected and credible intervals of parameters are therefore wide. The credible interval includes the true value for all parameters except the relative infectivity of children. On day 30, with 80 cases detected, posterior means are always relatively close to the true simulation value although credible intervals remain wide for some parameters like the relative infectivity and susceptibility of children and the mean generation time. On day 40 (182 cases detected), we would rightly conclude that children are more infectious and susceptible than adults although here again the credible intervals remain relatively wide. Properly characterizing the infectivity profile requires substantially more data (849 cases detected by day 60). Only 10 days after control measures are implemented, fairly accurate estimation of the efficacy of interventions in different settings becomes possible.

**Figure 4.**
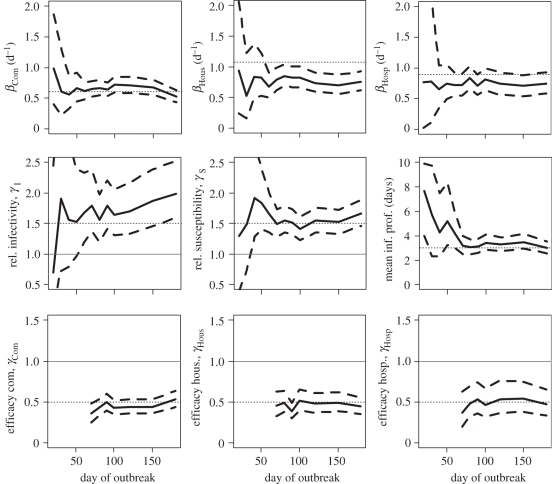
Estimates of transmission rates and relative transmission risk factors as a function of the number of days since the outbreak started when 50% of cases are detected in the community and in the hospital, and when 90% of cases among household contacts of detected cases are detected. Solid line, posterior mean; dashed line, 95% credible interval; dotted line, simulation value. For parameters used to compare groups (e.g. relative susceptibility, efficacy of interventions, etc.), we have also added a thin horizontal line *y* = 1. Top row gives estimates of the transmission rates in the different settings. Middle row gives estimates of the relative infectivity and relative susceptibility of children and the mean duration characterizing the infectivity profile. Bottom row gives the estimates of the efficacy of intervention to reduce transmission rates in the different settings.

When only 50 per cent of cases in the community and in hospitals are detected, performance of the approach remains satisfying although as expected credible intervals are wider and it takes longer for accuracy to be acceptable (figure 4). When 25 per cent of cases in the community and in hospitals are detected, precision of estimates starts to break down (electronic supplementary material, figure S1). Although estimates of transmission rates in the community and the hospital are not strongly affected by under-reporting in those settings, this is not true of estimates of transmission rates in small closed settings such as households (electronic supplementary material, figure S2).

**Figure 5.**
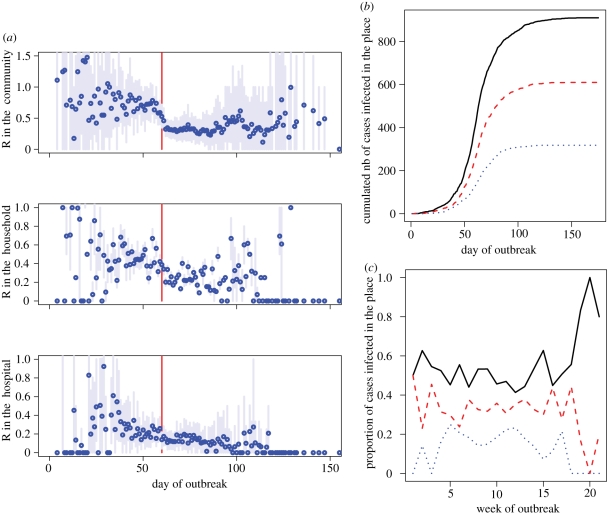
Summary statistics derived from the tree reconstruction. (*a*) Disaggregated monitoring of the reproduction number in the community, the household and the hospital based on the reconstructed transmission tree. Blue point, posterior mean; light blue line, 95% credible interval; red line, time when control measures were implemented. (*b*) Reconstructed cumulated number of cases infected in the different settings. (*c*) Reconstructed weekly proportion of cases infected in the different settings. (*b*,*c*) Solid line, community; dashed line, household; dotted line, hospital.

The method also allows disaggregated monitoring of the reproduction number and the number of cases infected in different settings (figure 5).

## 8. Discussion

In this paper, we have presented strategies to tackle some of the challenges associated with the estimation of transmission characteristics of infectious diseases and the risk factors affecting transmission patterns.

The dependency that is typically observed in outbreak data (i.e. the risk of infection for an individual depends on the infection status of other individuals) can potentially lead to long computational times. We showed that if risk factors are discretized, the inferential problem can be simplified to the analysis of (i) a dataset on cases only and (ii) a pre-compiled summary table on interactions between individuals of the population and cases. In the FMD application, discretization reduced the computational time from few weeks to few minutes with no change in the estimates of the transmission kernel. It is likely that with substantial effort and parallel programming, we could have reduced the computation time of brute force approach by one or two orders of magnitude. Even so, it seems unlikely that computational times could have gone much below few days. This has to be compared with the few minutes needed to run our algorithms on the FMD dataset. For small datasets, discretization may provide no computational gain if it takes longer to explore the set ***Ψ*** of transmission risk factors than to sum over the pairs {case *i*, individual *j*}. Discretization may be difficult to implement on particularly complex transmission models [19,23] in which case brute force calculation of the likelihood may be needed.

We presented two strategies to perform parameter estimation and tree reconstruction in a coherent way. The first one is a sequential approach that we used to analyse data from an influenza school outbreak [7]. Here, we implemented an alternative strategy where transmission parameters and the transmission tree are estimated simultaneously. There are pros and cons for each of those strategies. A nice feature of simultaneous inference is that the information on the transmission tree can sometimes lead to very simple and fast maximization routines in the frequentist setting (see appendix A) and to very good mixing in the MCMC chains in the Bayesian setting. However, for small datasets, we sometimes observed convergence problems in the Bayesian implementation of simultaneous inference. For example, let assume that there are two types *A* and *B* of individuals and that at iteration *i* of the MCMC, by chance, all the source cases in the transmission tree are of type *A*. If that happens, the chain may then be stuck at a local maximum where the infectivity of cases of type *B* is very close to 0. The EM algorithm does not suffer from that problem on small datasets; but has the disadvantage that it cannot be used when dates of infection are missing. The sequential Bayesian approach presented in Cauchemez *et al*. [7] may therefore be the most robust strategy, as it can be used in small datasets and when dates of infection are missing; but it requires extra work to tune the variance of the Metropolis-Hastings proposals to ensure satisfactory mixing.

For situations when the dates of infection are unknown, we presented models which assumed that infectivity depends on the time elapsed since symptom onset, and is independent of the time of infection. This assumption reduces the computational burden since it implies that the infection hazard an individual is exposed to solely depends on measured quantities plus the parameters of the model, rather than on the unobserved days of infection of other cases. In practice, assumption H1 seems acceptable for a relatively wide range of diseases since infectivity is often triggered or influenced by symptoms. However, where the assumption is invalid, more computationally intensive methods accounting for the unobserved times of infection become necessary [23].

In the simulation study, we considered an epidemic for which it would be possible to detect and identify a substantial proportion of cases; this would therefore be more applicable to a SARS-like scenario rather than pandemic influenza. The simulation study showed that even in situations where under-reporting is substantial (e.g. 50%), it would still be possible to obtain informative estimates of key characteristics. We found that estimates of the transmission rate in the community and in the hospital were relatively robust to under-reporting in those settings. This can be explained by the fact that in a large population, the exponential growth rate of the epidemic is not affected by under-reporting. But estimates of transmission rates in small social units such as households were—as might be expected—strongly affected by under-ascertainment of cases. Estimates of relative infectivity were particularly sensitive to under-reporting, with large variance and sometimes important bias. Estimating relative infectivity is in general quite challenging because it requires that one can compare the offspring of one group of individuals (e.g. adults) with that of another group (e.g. children) and this becomes very difficult as under-reporting increases. It is likely that estimates would be less robust to underreporting, if reporting rates changed over time, as probably happens in real epidemics.

Here, we have presented relatively simple approaches to reduce computational burden in the estimation of transmission parameters and to integrate parameter estimation and tree reconstruction in a coherent way. However, the analysis of outbreak data is subject to many other challenges. For example, it may be difficult to infer which parametric distribution should be used for the infectivity profile and the incubation period; data augmentation strategies may fail in a context when data are not missing at random or when there are false-positives or false-negatives [23]. A particular challenge in outbreak data is that they are rarely informative about the incubation period of the disease. In the simulations study, for example, we made the assumption that the distribution of the incubation period of the disease was known. If it was not the case, one would require extra data to estimate it. For example, in the past, data from outbreaks in an aeroplane [24] or in a bus [25] were used to estimate the incubation period of influenza.

A key practical challenge to implement the methods presented here in real time is the rapid collection and digitization of sufficiently detailed epidemiological data. However, recent experience demonstrates that it is possible to collect very detailed epidemiological data even during large outbreaks [10,13–15,25,26]. Cleaning and processing those data so that they are ready for analysis close to real-time remain a huge challenge, but the recent examples of the 2001 FMD outbreak in the UK [13,14], the 2003 SARS epidemic [15,26] and the 2009 H1N1 pandemic [25] show that this is increasingly feasible. However, reporting delays should always be expected and it will be important to account for those delays in future developments of the statistical method presented here.

Last, a key limit on the more widespread use of the type of methods presented in this paper is the relatively high technical hurdle to implement them, given there is currently no user-friendly statistical software package that allows easy implementation of this type of analysis. Developing such tools is therefore a priority.

## Appendix A

### A.1. Link between the continuous and the discrete time transmission models

We explain here the link between the continuous time and discrete time transmission models. At any (continuous) time point *u* during day *t* (i.e. *u* ∈ [*t*;*t* + 1)), the instantaneous hazard of infection exerted on individual *j* is

where *λ*_*j*_(*t*) is defined in the main text. So, we make the assumption that the instantaneous hazard of infection is a step function with daily steps. Conditional on the fact that individual *j* has not been infected up to day *t*, the probability that individual *j* is infected on day *t* is equal to

In the continuous time model, conditional on the (continuous) time of infection *u*_*j*_ of case *j* with *u*_*j*_ ∈ [*t*_*j*_; *t*_*j*_ + 1), the probability that case *i* is the case source is:

And we note that this probability is constant for *u*_*j*_ ∈ [*t*_*j*_; *t*_*j*_ + 1).

Conditional on the day *t*_*j*_ of infection, the probability that case *i* is the case source given in equation (4.1) is:

and

### A.2. Simplified routines for relative risks in a context of small forces of infection

Let consider the common situation where

— we are interested in relative risks (i.e. comparison with a reference group). For example, the population is partitioned in *C*_*k*_ groups  on the basis of risk factor *k* so that the multiplicative term associated with risk factor *k* in equation (2.4) is:
— the force of infection exerted on individuals is relatively small so that the log-likelihood simplifies to equation (4.4):

For the conditional maximization of the likelihood with respect to  in the ECM algorithm (box 1), the log-likelihood can be re-written:

where *A* is a term that does not depend on . So, the maximum for parameter  conditional on other parameters is simply:

In the Bayesian setting, if we assume that parameter  has a gamma prior *Γ*(*a,b*), a Gibbs sampler update [1] for parameter  is possible in the MCMC with:

### A.3. Derivation of probabilities *P*_j_*(t)*

Let *U*_*t*_ = {*t*_*i*_; *t*_*i*_< *t*} and *S*_*t*_ = {*s*_*i*_; *t*_*i*_< *t*}, respectively, denote the dates of infection and of symptoms onset of cases infected before day *t*. Hypothesis H1 implies that:

so that one only needs the sequence of symptom onset dates to reconstruct the risk individual *j* was exposed to

and

Given {*s*_*j*_, *S*_*t*_}, the probability that case *j* is infected on day *t* is therefore:
